# Minimally Invasive Aortic Valve Replacement and Perioperative Stroke Risk: A Propensity-Matched Analysis

**DOI:** 10.3390/jcm15207803

**Published:** 2026-10-10

**Authors:** Adam Ryszard Kowalówka, Julia Wojtczak, Maria Zwinczak, Barbara Wawrzyńczak, Mikołaj Jodłowski, Radosław Gocoł

**Affiliations:** 1 Department of Cardiac Surgery, Upper-Silesian Heart Center, 40-635 Katowice, Poland; 2 Department of Cardiac Surgery, Medical University of Silesia, 40-055 Katowice, Poland; juliawojtczak522@wp.pl (J.W.); zwinczakmaria@gmail.com (M.Z.);

**Keywords:** aortic valve replacement, minimally invasive surgery, stroke, transient ischemic attack, propensity score matching

## Abstract

**Background/Objectives:** Stroke is a devastating complication after aortic valve replacement (AVR). Whether minimally invasive AVR (miniAVR) reduces perioperative stroke risk versus conventional sternotomy remains uncertain. **Methods:** This single-center retrospective cohort study used propensity score matching (PSM) in 1741 consecutive patients undergoing isolated AVR (2014–2025). After exclusions, 1:1 PSM using 10 covariates created 155 matched pairs with excellent balance (all standardized mean differences < 0.20). The primary endpoint was in-hospital stroke or transient ischemic attack (TIA); secondary endpoints were 30-day and 5-year mortality and major complications. Firth penalized logistic regression identified independent predictors of stroke/TIA. **Results:** Stroke/TIA occurred in 0/155 (0.0%) miniAVR versus 7/155 (4.5%) conventional patients (*p* = 0.015); the rule-of-three upper 95% limit for miniAVR was ~1.9%. Thirty-day mortality was similar (0.6% vs. 2.6%; *p* = 0.371). Five-year survival favored miniAVR (94.6% vs. 87.2%; log-rank *p* = 0.047). miniAVR was independently associated with lower stroke/TIA odds (odds ratio [OR] 0.052; 95% confidence interval [CI] 0.003–0.832; *p* = 0.037); longer cardiopulmonary bypass time was associated with higher odds (OR 1.014/minute; 95% CI 1.001–1.028; *p* = 0.032). **Conclusions:** miniAVR was associated with zero observed perioperative strokes and higher 5-year survival; however, this survival comparison is limited by short median follow-up in the miniAVR group (1.9 years). Given the small number of events and the single-center design, these findings should not be interpreted as proof of a causal neuroprotective effect. Multicenter validation with longer, balanced follow-up is required.

## 1. Introduction

Aortic stenosis (AS) is the most prevalent valvular heart disease in adults and the leading indication for valve intervention in the aging population, with a substantial and rising burden documented in contemporary registries [1]. According to the 2025 European Society of Cardiology/European Association for Cardio-Thoracic Surgery (ESC/EACTS) Guidelines for the management of valvular heart disease, treatment decisions should be individualized by the Heart Team, balancing patient age, anatomy, comorbidities, and preferences [2]. The updated guidelines favor transcatheter aortic valve implantation (TAVI) over surgical aortic valve replacement (SAVR) in older patients, while SAVR remains the standard option for younger patients and those with anatomical features unfavorable for a transcatheter approach [2]. Surgical aortic valve replacement (AVR) therefore continues to be performed in large numbers worldwide.

Stroke remains one of the most devastating complications following cardiac surgery and a critical determinant of postoperative morbidity, mortality, and long-term functional outcomes. In isolated SAVR, the reported incidence of perioperative stroke is generally in the low single-digit percentage range, depending on patient risk profile and diagnostic criteria [3,4,5]. In contemporary low-risk surgical cohorts, stroke rates of approximately 1–2% have been reported [6]. The pathophysiology of perioperative stroke is multifactorial, involving atheromatous embolization during aortic cannulation and cross-clamping, air embolism, hypoperfusion during cardiopulmonary bypass (CPB), and postoperative atrial fibrillation (AF). Given that stroke after SAVR markedly increases in-hospital mortality and is associated with prolonged intensive care unit stays and diminished quality of life, strategies to reduce cerebrovascular complications represent a paramount objective in contemporary cardiac surgery.

Minimally invasive aortic valve replacement (miniAVR) has evolved over the past two decades as an alternative to conventional full sternotomy, aiming to reduce surgical trauma while maintaining equivalent hemodynamic outcomes [7,8]. The most widely adopted techniques include upper partial sternotomy and right anterior mini-thoracotomy [3,9]. A recent network meta-analysis comparing minimally invasive techniques confirmed favorable perioperative profiles across the spectrum of minimally invasive accesses [10], and a systematic review and meta-analysis comparing mini-sternotomy with right anterior mini-thoracotomy reported comparable safety with technique-specific trade-offs [11]. Minimally invasive approaches have also been associated with lower resource utilization after left-heart valve surgery [12]. In many high-volume centers, miniAVR has become a standard procedure for isolated aortic valve disease [7,8].

Despite the growing body of evidence supporting the safety of miniAVR, data specifically addressing its impact on perioperative stroke incidence remain scarce and inconclusive [10,11]. The theoretical basis for a potential neuroprotective effect rests on several mechanistic considerations: reduced manipulation of the ascending aorta may decrease atheromatous embolization; comparable or shorter CPB times may limit non-pulsatile perfusion; and a diminished systemic inflammatory response may attenuate endothelial dysfunction [7,8]. Therefore, the aim of the present study was to compare the incidence of perioperative stroke or transient ischemic attack (TIA) between miniAVR via upper hemisternotomy and conventional AVR via full median sternotomy in a propensity score-matched cohort, and to identify independent predictors of this complication.

## 2. Materials and Methods

### 2.1. Study Objective, Design, and Population

The objective of this study was to determine whether miniAVR via upper hemisternotomy is associated with a lower incidence of perioperative stroke or TIA than conventional AVR via full median sternotomy, and to identify independent predictors of this complication.

The study was designed according to the following PICO(T) framework (Population, Intervention, Comparator, Outcomes, Timing):

- Population (P): Adult patients (age ≥ 18 years) undergoing elective, isolated aortic valve replacement for predominant aortic stenosis or regurgitation.
- Intervention (I): Minimally invasive aortic valve replacement via upper hemisternotomy (miniAVR).
- Comparator (C): Conventional aortic valve replacement via full median sternotomy.
- Outcomes (O): Primary—composite of perioperative stroke or transient ischemic attack (TIA); Secondary—30-day mortality, 5-year survival, and major postoperative complications.
- Timing (T): January 2014 to December 2025.

We hypothesized that miniAVR would be associated with a significantly lower incidence of perioperative stroke/TIA compared with conventional sternotomy, with comparable safety and survival outcomes. To minimize selection bias, we employed 1:1 propensity score matching with 10 baseline covariates. Independent predictors of perioperative stroke or TIA were assessed using multivariable Firth penalized logistic regression analysis.

**Figure 1:**
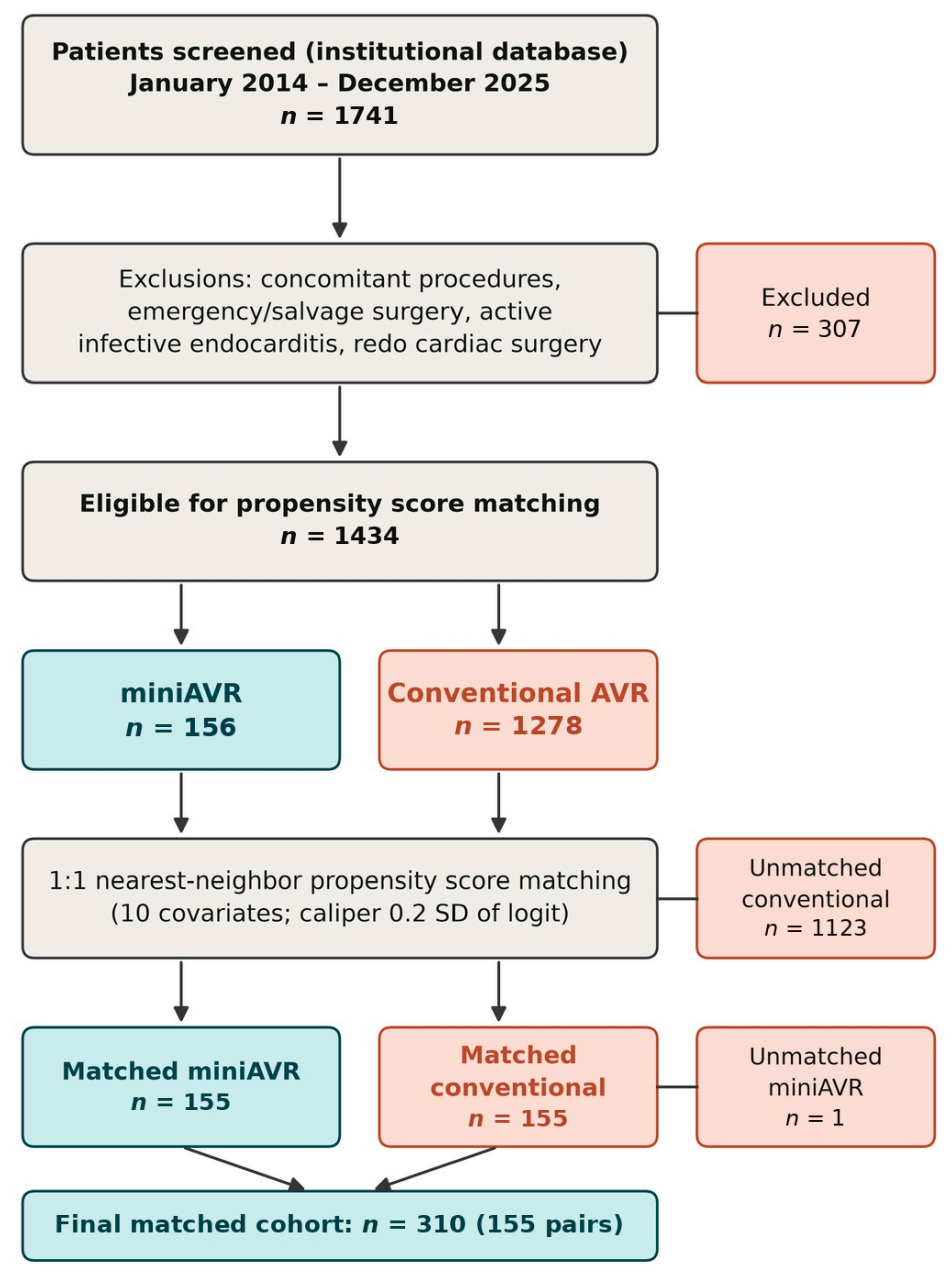
STROBE patient-selection flow diagram. From 1741 screened patients, 1434 remained eligible after exclusions (156 miniAVR and 1278 conventional; Supplementary Table S1); 1:1 propensity score matching yielded 155 matched pairs (310 patients).

This is a single-center, retrospective, observational cohort study with propensity score matching. We screened all consecutive adult patients (≥18 years) who underwent isolated, elective surgical aortic valve replacement at our institution between January 2014 and December 2025. Patients undergoing concomitant procedures (coronary artery bypass grafting, mitral or tricuspid surgery, aortic root or ascending aortic replacement), emergency or salvage operations, active infective endocarditis, or redo cardiac surgery were excluded. Eligible patients treated with miniAVR via upper hemisternotomy were compared with those treated with conventional AVR via full median sternotomy. The patient-selection process is presented in the Strengthening the Reporting of Observational Studies in Epidemiology (STROBE) flow diagram (Figure 1).

Emergency and salvage operations were excluded to avoid confounding by preoperative anoxic brain injury. Patients requiring emergency or salvage surgery frequently present with hemodynamic instability, cardiogenic shock, preoperative cardiopulmonary resuscitation, or the need for mechanical ventilation and inotropic support; the resulting cerebral hypoperfusion and hypoxemia may cause hypoxic–ischemic (anoxic) brain injury before the operation begins. Such injury independently predisposes to postoperative neurological deficits, is difficult to distinguish clinically and radiologically from a new perioperative stroke, and is unrelated to the surgical access. Moreover, because these patients are typically operated on via full sternotomy, their inclusion would have systematically increased the stroke risk in the conventional group and biased the comparison in favor of miniAVR. Redo cardiac surgery was excluded for a similar reason: resternotomy, adhesiolysis, and manipulation of a previously operated ascending aorta carry a distinct embolic risk profile and require different access and cannulation strategies, which would have introduced heterogeneity unrelated to the comparison of interest. Restricting the cohort to elective, first-time isolated AVR therefore reduced confounding by indication and selection bias, at the cost of generalizability.

The minimally invasive AVR program was introduced and progressively expanded at our institution during the study period. Consequently, most miniAVR procedures were performed in the later years of the study period, which explains the shorter follow-up in the miniAVR group.

### 2.2. Ethics Statement

The study was conducted in accordance with the Declaration of Helsinki (2013 revision). Institutional Review Board was consulted, and patient consent was waived (PCN/CBN/0022/KB/214/21; 12 October 2021). Baseline clinical and procedural data, and outcomes at follow-up were entered into prespecified electronic case report forms. Follow-up status was assessed by personal contact or from the National Health Fund.

### 2.3. Surgical Technique

In the miniAVR group, the aortic valve was accessed through an upper partial (J-shaped) hemisternotomy extending into the third or fourth right intercostal space. Cardiopulmonary bypass was established by central or peripheral cannulation according to surgeon preference and patient anatomy. In the conventional group, a standard full median sternotomy with central cannulation was performed. In both groups, myocardial protection was most commonly achieved with Del Nido cardioplegia. Choice of prosthesis (biological or mechanical) followed current guideline recommendations and shared decision-making [2]. All procedures were performed by experienced consultant surgeons.

### 2.4. Data Collection and Endpoints

Demographic, clinical, echocardiographic, intraoperative, and postoperative data were extracted from the institutional cardiac surgery database. Long-term follow-up, including vital status and date of death, was not based on outpatient clinic visits; these data were obtained from the national health database of the National Health Fund, which includes information on patient deaths. The primary endpoint was the composite of in-hospital ischemic stroke or transient ischemic attack (TIA), defined according to standard clinical and radiological criteria. Secondary endpoints comprised 30-day mortality, overall and 5-year survival, postoperative atrial fibrillation, acute renal failure, re-exploration for bleeding or tamponade, permanent pacemaker implantation, duration of mechanical ventilation, intensive care unit (ICU) stay, and total hospital length of stay. Cardiopulmonary bypass (CPB) and aortic cross-clamp times were recorded for all patients.

### 2.5. Propensity Score Matching

To minimize confounding by indication, 1:1 propensity score matching was performed. The propensity score was estimated by multivariable logistic regression with miniAVR as the dependent variable and the following 10 baseline covariates: age, sex, body mass index, left ventricular (LV) ejection fraction, estimated glomerular filtration rate (GFR), diabetes mellitus, arterial hypertension, atrial fibrillation, bicuspid aortic valve morphology, and predominant valve lesion. Nearest-neighbor matching without replacement was applied using a caliper of 0.2 standard deviations of the logit of the propensity score. Matching quality was assessed by standardized mean differences (SMD), with values < 0.20 considered indicative of adequate balance. Duration of follow-up was not used as a matching variable because it is not a baseline patient characteristic but is determined by the calendar date of surgery.

### 2.6. Statistical Analysis

Continuous variables are presented as mean ± standard deviation or median (interquartile range, IQR) according to distribution, assessed by the Shapiro–Wilk test. Categorical variables are expressed as counts and percentages; the diagnostic categories of severe aortic stenosis and at-least-moderate aortic regurgitation are not mutually exclusive, as patients may present with mixed aortic valve disease, so their percentages in Table 1 do not sum to 100%. In the matched cohort, between-group comparisons used the independent-samples *t*-test or Mann–Whitney U test for continuous variables and the chi-square or Fisher exact test for categorical variables, as appropriate; matched pairs were treated as independent groups for these comparisons because greedy nearest-neighbor matching without replacement does not preserve a 1:1 correspondence suitable for paired analysis once unmatched candidates are discarded. Because the primary endpoint occurred in zero patients in the miniAVR group, conventional logistic regression was subject to complete separation. We therefore used Firth penalized maximum likelihood logistic regression, which reduces small-sample bias and yields finite estimates under separation [13,14]. For the zero-event group, the upper bound of the 95% confidence interval (CI) was additionally approximated using the rule of three [15].

Given the rarity of the primary event, the multivariable Firth model was restricted to three pre-specified covariates (surgical approach, CPB time, and age) to maintain an acceptable events-per-variable ratio and to avoid overfitting; this analysis is explicitly exploratory and hypothesis-generating. Survival was analyzed by the Kaplan–Meier method with the log-rank test, and restricted mean survival time (RMST) at 5 years was computed as a complementary, assumption-robust measure. A two-sided *p*-value < 0.05 was considered statistically significant. Analyses were performed in IBM SPSS Statistics (v. 28) and Python ver. 3.14.8 (statsmodels, lifelines).

## 3. Results

### 3.1. Study Population and Propensity Score Matching

**Figure 2:**
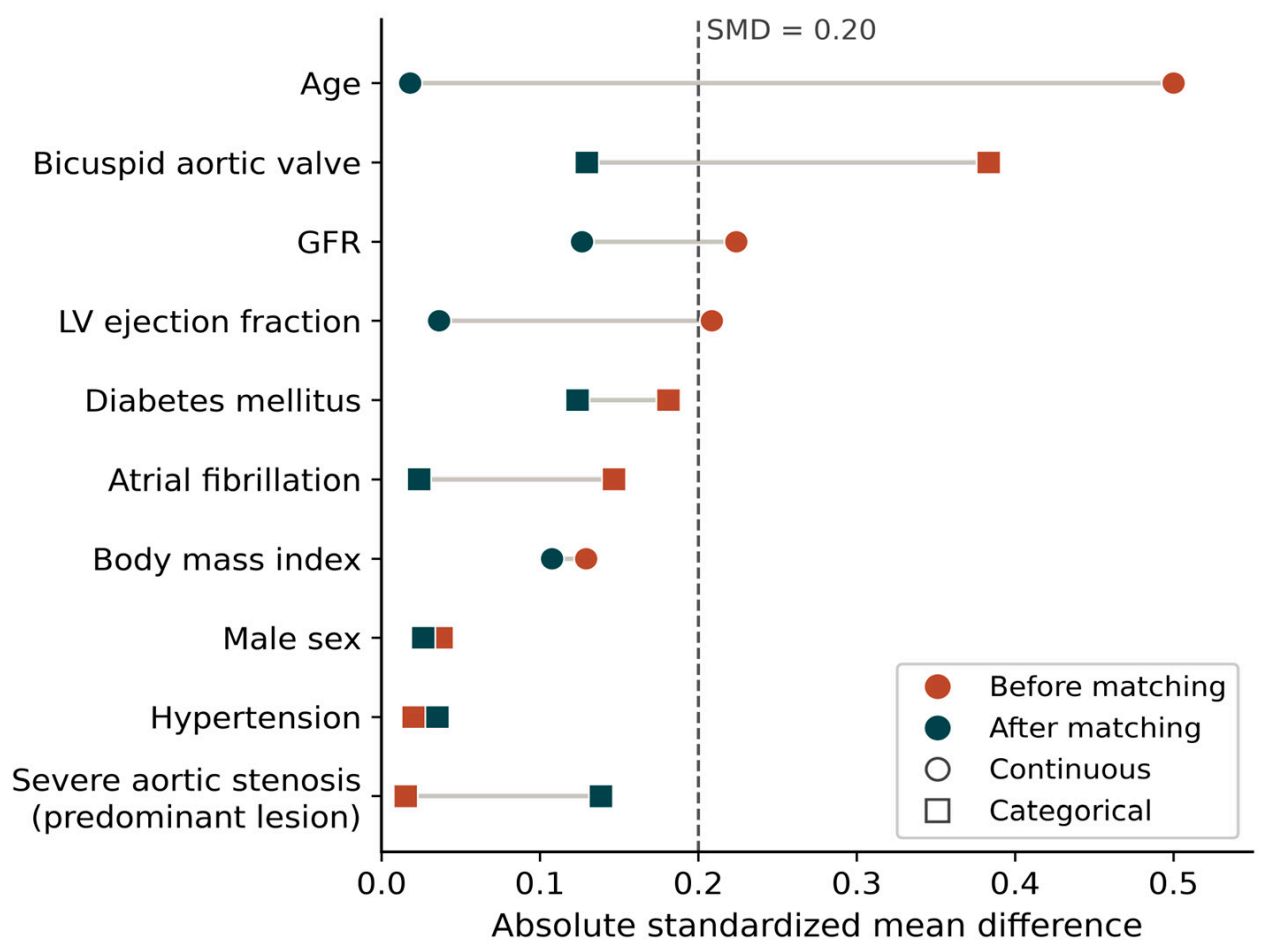
Love plot of standardized mean differences (SMD) before (red) and (green) after propensity score matching. All 10 matching covariates achieved SMD < 0.20 after matching, indicating excellent balance consistent with the non-significant between-group *p*-values in Table 1.

Of the 1741 screened patients, 307 met at least one exclusion criterion, leaving 1434 eligible patients (156 miniAVR and 1278 conventional; Figure 1); their baseline characteristics before matching are presented in Supplementary Table S1. Propensity score matching yielded 155 matched pairs (310 patients); one miniAVR patient could not be matched within the specified caliper and was excluded from the matched analysis (Figure 1). After matching, the standardized mean differences (SMD) of all 10 matching covariates were below 0.20 (Figure 2), and the propensity score distributions overlapped substantially (Supplementary Figure S1). Matched patients had a mean age of 66.1 ± 10.5 years (miniAVR) and 65.9 ± 11.8 years (conventional; *p* = 0.853), and the two groups were comparable with respect to sex, body mass index, LV ejection fraction, renal function, and prevalence of bicuspid aortic valve morphology (Table 1). Peripheral vascular disease, which was not among the 10 matching covariates, remained imbalanced after matching (12.3% vs. 3.9%; *p* = 0.006; Table 1).

### 3.2. Operative and Perioperative Data

Cardiopulmonary bypass time was modestly longer in the miniAVR group (median 70 min, IQR 55–88) than in the conventional group (64 min, IQR 54–75; *p* = 0.010), whereas aortic cross-clamp time did not differ significantly (52 vs. 50 min; *p* = 0.378). Total operative time was comparable between groups (165 vs. 180 min; *p* = 0.054). Biological prostheses were used more frequently in the miniAVR group (91.6% vs. 63.9%; *p* < 0.001). Del Nido cardioplegia was employed in 90.3% of miniAVR and 81.9% of conventional procedures (*p* = 0.010). Postoperative mechanical ventilation was shorter after miniAVR (median 8.4 h, IQR 6.7–12.7 vs. 10.7 h, IQR 8.0–13.6; *p* = 0.002), while median ICU stay (51.9 h, IQR 46.6–75.6 vs. 54.3 h, IQR 49.5–76.2; *p* = 0.099) and total hospital length of stay (7 days, IQR 7–8 vs. 7 days, IQR 7–9; *p* = 0.610) were similar (Table 2 and Supplementary Table S3).

### 3.3. Primary Endpoint: Perioperative Stroke/TIA

**Figure 3:**
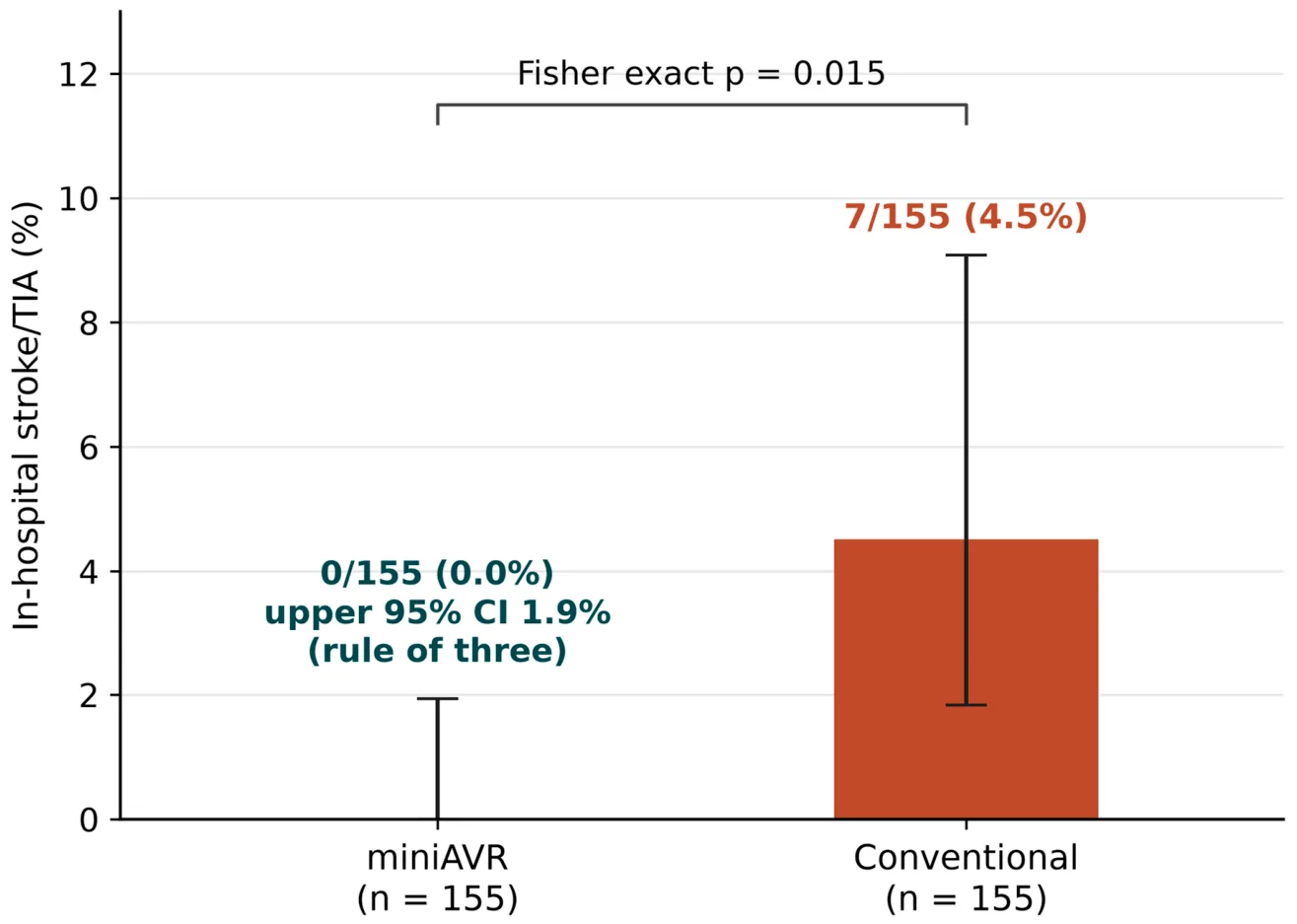
Incidence of the primary composite endpoint (in-hospital stroke or TIA). miniAVR 0/155 (0.0%) versus conventional 7/155 (4.5%); Fisher exact *p* = 0.015. TIA, transient ischemic attack. Error bars indicate the exact (Clopper–Pearson) 95% CI for the conventional group and the rule-of-three upper 95% confidence limit (1.9%) for the miniAVR group.

The primary composite endpoint of in-hospital stroke or TIA occurred in 0 of 155 miniAVR patients (0.0%) versus 7 of 155 conventional patients (4.5%; Fisher exact *p* = 0.015). This corresponded to an absolute risk difference of 4.5%. Because no events occurred in the miniAVR group, the upper bound of the 95% confidence interval for the stroke/TIA rate was estimated at approximately 1.9% using the rule of three [15]. The Haldane–Anscombe corrected odds ratio (OR) was 0.06 (95% CI 0.004–1.12) (Figure 3).

### 3.4. Secondary Outcomes

Thirty-day mortality was numerically lower after miniAVR (1/155, 0.6%) than after conventional surgery (4/155, 2.6%), but the difference was not statistically significant (relative risk 0.25; *p* = 0.371). Overall mortality across the entire available follow-up period (median 1.9 years for miniAVR and 4.3 years for conventional; see Section 3.5) was 7/155 (4.5%) for miniAVR versus 17/155 (11.0%) for conventional surgery (*p* = 0.054; Table 3). Major postoperative complications were comparable between groups, including postoperative atrial fibrillation (13.5% vs. 10.3%; *p* = 0.484), acute renal failure (1.3% vs. 1.3%), re-exploration for bleeding or tamponade (1.3% vs. 1.9%), and permanent pacemaker implantation (0.6% vs. 2.6%; *p* = 0.371). Pleural drainage volume and postoperative troponin did not differ significantly between groups (Table 3 and Supplementary Table S3).

### 3.5. Survival

**Figure 4:**
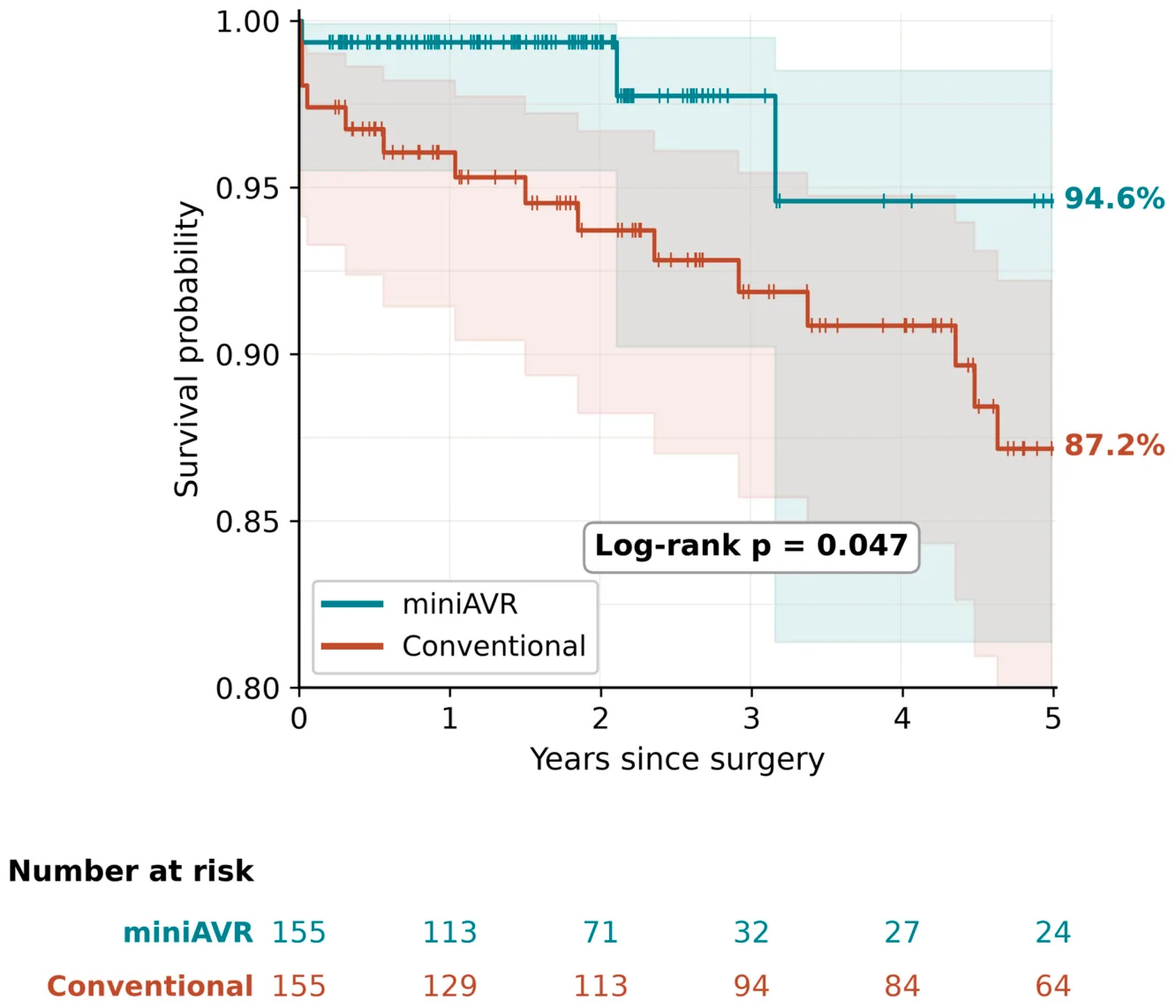
Kaplan–Meier survival curves over 5 years. Five-year survival was 94.6% (miniAVR) versus 87.2% (conventional); log-rank *p* = 0.047. Shaded areas indicate 95% CIs. The number-at-risk table is provided in Supplementary Table S2. Because median follow-up was substantially shorter in the miniAVR group (1.9 vs. 4.3 years), 5-year estimates are based on a small subset of early-treated patients in each group and should be interpreted with caution.

Over a median follow-up of 1.9 years (IQR 0.9–2.7) in the miniAVR group and 4.3 years (IQR 1.8–7.3) in the conventional group, Kaplan–Meier estimated survival was higher after miniAVR. Estimated survival at 1, 3, and 5 years was 99.4%, 97.8%, and 94.6% for miniAVR versus 96.1%, 91.9%, and 87.2% for conventional surgery (log-rank χ^2^ = 3.934; *p* = 0.047). The 5-year restricted mean survival time favored miniAVR (4.86 vs. 4.65 years; difference +0.21 years). Within 5 years of surgery, 3 deaths (1.9%) occurred in the miniAVR group versus 15 (9.7%) in the conventional group (*p* = 0.006) (Figure 4). Only 24 miniAVR patients (15.5% of the matched miniAVR cohort) and 64 conventional patients (41.3%) remained at risk at 5 years (Supplementary Table S2).

### 3.6. Independent Predictors of Stroke/TIA

Because the primary endpoint exhibited complete separation (zero events in the miniAVR group), independent predictors were assessed using Firth penalized logistic regression [13,14]. In the pre-specified three-covariate multivariable model, miniAVR was independently associated with markedly lower odds of stroke/TIA (OR 0.052; 95% CI 0.003–0.832; *p* = 0.037), corresponding to approximately 95% lower odds relative to conventional surgery. Longer CPB time was associated with higher odds of stroke/TIA (OR 1.014 per minute; 95% CI 1.001–1.028; *p* = 0.032), whereas age was not a significant predictor (OR 0.975; 95% CI 0.923–1.030; *p* = 0.369). The model showed acceptable fit (Akaike information criterion [AIC] 61.17; McFadden pseudo-R^2^ 0.205; Hosmer–Lemeshow χ^2^ = 5.16; *p* = 0.523; full diagnostics in Supplementary Table S4), although the low events-per-variable ratio (2.3) underscores the exploratory nature of this analysis (Table 4). Univariable Firth estimates are presented in Table 5, and the consistency of the stroke/TIA risk reduction with miniAVR across clinically relevant subgroups (age, LV ejection fraction, preoperative atrial fibrillation, and renal function) is illustrated in Supplementary Figure S2.

## 4. Discussion

In this single-center, propensity score-matched cohort of 310 patients undergoing isolated surgical aortic valve replacement, miniAVR via upper hemisternotomy was associated with no observed perioperative strokes or TIAs, compared with a 4.5% incidence after conventional full sternotomy (*p* = 0.015), together with numerically higher 5-year survival (94.6% vs. 87.2%; *p* = 0.047), although the latter comparison is limited by markedly unequal follow-up duration between groups. After accounting for the complete separation inherent to a zero-event group, miniAVR remained independently associated with lower stroke/TIA odds in an exploratory Firth-penalized model (OR 0.052; 95% CI 0.003–0.832; *p* = 0.037). Given the rarity of the primary event, the single-center design, and the wide confidence intervals, these findings should be regarded as a trend rather than proof of a causal neuroprotective benefit of miniAVR, and they add to the growing evidence that minimally invasive approaches can be performed safely without compromising neurological outcomes [7,10,11].

Several aspects of these findings require cautious interpretation. The absolute risk difference of 4.5% in stroke/TIA should not be translated into a directly actionable number-needed-to-treat: with zero events in one arm and very few in the comparator, this observational estimate is imprecise, and no causal treatment effect can be inferred. The survival findings must likewise be interpreted in light of the markedly different follow-up between groups (median 1.9 vs. 4.3 years), which reflects the progressive introduction of the minimally invasive program at our institution, with most miniAVR procedures performed in the later part of the study period. Consequently, only 24 miniAVR patients (15.5%) and 64 conventional patients (41.3%) remained at risk at 5 years, and the 5-year estimates for miniAVR are based on a small, earlier-treated subgroup that may be subject to immortal-time and cohort-maturity bias. For the same reason, the cumulative mortality over the entire follow-up (Table 3) and the number of deaths within 5 years describe different time windows and should not be compared directly: of the 7 deaths in the miniAVR group, 3 occurred within the first 5 years and the remaining 4 later, among the small subset of patients with longer accrued follow-up. These survival estimates should therefore be regarded as preliminary and require confirmation once longer, more balanced follow-up accrues.

### 4.1. Comparison with Prior Literature

Our results are consistent with the contemporary body of evidence on minimally invasive AVR. A recent state-of-the-art review of minimally invasive cardiac surgery emphasized that reduced surgical trauma and preserved sternal integrity may translate into faster recovery and fewer systemic complications [7]. A network meta-analysis of minimally invasive techniques found favorable perioperative profiles across accesses [10], and a systematic review and meta-analysis comparing mini-sternotomy with right anterior mini-thoracotomy reported comparable safety with technique-specific trade-offs [11]. Several propensity-matched and large comparative series—including the anterior right minithoracotomy cohort reported by Bowdish and colleagues [3], the 1130-patient comparison of mini-sternotomy and mini-thoracotomy by Fattouch et al. [4], and the three-way comparison of conventional, mini-thoracotomy, and mini-sternotomy approaches by Shen et al. [16]—have reported perioperative outcomes that are at least equivalent to conventional sternotomy.

Single-center and registry analyses likewise support these observations. Liu and colleagues reported favorable propensity-matched outcomes for mini-sternotomy versus full sternotomy [5], and Dumani et al. [17] and Karic et al. [18] documented comparable safety with potential advantages in recovery for the minimally invasive approach. Long-term data are also reassuring: a 10-year comparison of mini-sternotomy and full sternotomy demonstrated durable outcomes [19], and minimally invasive techniques have been applied successfully even in higher-risk settings such as reoperative aortic valve surgery [20] and other high-risk patient groups [8]. Importantly, minimally invasive surgery has been associated with lower resource utilization after left-heart valve surgery [12], an increasingly relevant consideration for healthcare systems.

With respect to the choice between surgical and transcatheter therapy, our institutional analysis of the Aortic Valve Multicenter Registry demonstrated that SAVR remains a robust option in low-risk patients, with perioperative stroke rates in the low single-digit range [6]. The present finding of zero observed strokes after miniAVR therefore situates the minimally invasive surgical approach favorably within the contemporary treatment landscape outlined by the 2025 ESC/EACTS guidelines [2]. These results are also concordant with our earlier single-center experience with miniAVR [21]. A further recent analysis reinforces this picture: a 2025 meta-analysis of 75 comparative studies found that minimally invasive AVR was associated with lower 30-day mortality than conventional full-sternotomy AVR (OR 0.65, 95% CI 0.54–0.78) despite longer cross-clamp and cardiopulmonary bypass times [22], consistent with the neurological and perioperative safety signal observed in our cohort.

### 4.2. Potential Mechanisms

Several mechanisms may explain a lower perioperative stroke risk with miniAVR. The upper hemisternotomy approach permits central aortic cannulation under direct vision while limiting manipulation of the ascending aorta, which may reduce atheromatous embolization—a principal mechanism of perioperative ischemic stroke [7,8]. Refinements in operative conduct, including rapid-deployment and sutureless prostheses implanted through limited access [23], can streamline the procedure and offset the modestly longer CPB times observed in minimally invasive series; the comparable total operative time despite longer CPB time in our cohort may likewise reflect growing team experience and a maturing, standardized minimally invasive protocol that streamlined non-bypass operative steps over the study period. In our cohort, although CPB time was slightly longer in the miniAVR group, cross-clamp time was comparable, and longer CPB time itself emerged as an independent predictor of stroke/TIA, reinforcing the importance of operative efficiency [4,16]. In contrast, age was not independently associated with stroke/TIA in our exploratory model (OR 0.975; 95% CI 0.923–1.030; *p* = 0.369), although advanced age is a well-recognized risk factor for perioperative stroke after cardiac surgery. This finding most likely reflects the limited size of the study population and the small number of primary events (n = 7), which provided insufficient statistical power to detect a moderate effect of age. In addition, the matched cohort comprised relatively young (mean age 66 years), elective patients, and age was one of the matching covariates, which narrowed the age-related risk gradient. This null result should therefore not be interpreted as evidence that age is unrelated to perioperative stroke risk. Finally, a smaller incision and preserved chest-wall integrity may attenuate the systemic inflammatory response and shorten mechanical ventilation, as reflected by the reduced ventilation time observed in the miniAVR group [7,11].

### 4.3. Clinical Implications

If confirmed in larger multicenter studies, these findings would support broader adoption of miniAVR in suitable candidates with isolated aortic valve disease, particularly when neurological risk reduction is a priority. The associated improvements in ventilation time and resource utilization [12], together with comparable complication rates, suggest that minimally invasive AVR can be integrated into routine practice without an early-safety penalty when performed by experienced teams [7,11].

### 4.4. Limitations

This study has several important limitations. First, it is a single-center, retrospective, observational analysis, and despite rigorous propensity score matching, residual and unmeasured confounding cannot be excluded; notably, peripheral vascular disease was not among the 10 matching covariates and remained imbalanced after matching (Table 1), and this residual imbalance should be considered when interpreting between-group comparisons. Moreover, propensity score matching can only balance variables recorded in the institutional database; potentially relevant factors such as the degree of ascending aortic atherosclerosis or calcification, the operating surgeon, and the calendar year of surgery were not included in the matching model. Matching also retained only 155 of the 1278 eligible conventional patients (12.1%), and one miniAVR patient could not be matched; the matched cohort therefore represents a selected, lower-risk subset of the conventional population, which limits the generalizability of the results. Second, the primary endpoint was rare, with zero events in the miniAVR group, producing complete separation; although we addressed this with Firth penalized regression and the rule of three [13,14,15], the confidence intervals are wide, and the multivariable model—restricted to three covariates with an events-per-variable ratio of 2.3—is explicitly exploratory and hypothesis-generating. Relatedly, with only 7 primary events in total, the study had sufficient statistical power to detect only very large between-group differences in stroke/TIA risk and was underpowered to identify weaker predictors such as age; a true effect of more modest magnitude therefore cannot be excluded. Third, the difference in follow-up duration between groups was substantial (median 1.9 vs. 4.3 years), reflecting the progressive introduction of the minimally invasive program at our institution, and the longer follow-up in the conventional group may influence the survival comparison; in particular, 5-year survival estimates for miniAVR rely on a small subset of patients treated earliest in the study period, and immortal-time or cohort-maturity bias cannot be excluded. Follow-up duration could not be used as a matching variable, because it is determined by the date of surgery rather than by baseline patient characteristics. Fourth, prosthesis selection differed between groups, reflecting era and practice patterns. Fifth, the exclusion of emergency, salvage, and redo operations, although intended to avoid confounding by preoperative anoxic brain injury and by a distinct embolic risk profile, means that our findings apply only to elective, first-time isolated AVR. Finally, the results derive from a single high-volume center and may not be generalizable to all settings. Multicenter, prospective validation with longer and more balanced follow-up is warranted [10,11].

## 5. Conclusions

In this single-center, propensity score-matched cohort, minimally invasive aortic valve replacement was associated with no observed perioperative strokes or TIAs and with numerically higher 5-year survival compared with conventional full sternotomy, while major complication rates were comparable; the survival comparison is limited by unequal follow-up duration between groups. Given the exploratory nature of the analysis, the small number of primary events, and the single-center retrospective design, these findings do not establish a causal neuroprotective effect and require confirmation in larger, prospective, multicenter studies with balanced follow-up.

## Figures and Tables

**Table 1:** Baseline characteristics after propensity score matching.

Variable	miniAVR (*n* = 155)	Conventional (*n* = 155)	*p*-Value
Age, years	66.1 ± 10.5	65.9 ± 11.8	0.853
Male sex	89 (57.4%)	91 (58.7%)	0.910
Body mass index, kg/m^2^	28.1 ± 4.6	28.6 ± 4.7	0.270
LV ejection fraction, %	55.1 ± 8.6	55.4 ± 7.9	0.670
GFR, mL/min/1.73 m^2^	79.6 ± 13.7	81.3 ± 13.2	0.370
EuroSCORE	1.3 ± 0.9	1.3 ± 1.1	1.000
Hypertension	131 (84.5%)	129 (83.2%)	0.880
Diabetes mellitus	31 (20.0%)	39 (25.2%)	0.340
Atrial fibrillation (paroxysmal)	13 (8.4%)	12 (7.7%)	1.000
Cerebrovascular disease	3 (1.9%)	3 (1.9%)	1.000
Peripheral vascular disease ᵇ	19 (12.3%)	6 (3.9%)	0.006
Bicuspid aortic valve	79 (51.0%)	69 (44.5%)	0.306
Severe aortic stenosis ᵃ	133 (85.8%)	125 (80.6%)	0.290
Aortic regurgitation ≥ moderate ᵃ	52 (33.5%)	64 (41.3%)	0.200

Values are mean ± standard deviation or n (%). ^a^ Severe aortic stenosis and ≥moderate aortic regurgitation are not mutually exclusive categories (mixed aortic valve disease); percentages therefore do not sum to 100%. ^b^ Peripheral vascular disease was not among the 10 covariates used for propensity score matching (Section 2.5) and remained imbalanced after matching (SMD 0.31); this residual imbalance is noted as a limitation in Section 4.4. GFR, estimated glomerular filtration rate; LV, left ventricular.

**Table 2:** Operative and perioperative characteristics.

Variable	miniAVR (*n* = 155)	Conventional (*n* = 155)	*p*-Value
CPB time, min	70 (55–88)	64 (54–75)	0.010
Aortic cross-clamp time, min	52 (39–66)	50 (41–61)	0.378
Total operative time, min	165 (135–205)	180 (150–200)	0.054
Biological prosthesis	142 (91.6%)	99 (63.9%)	<0.001
Mechanical prosthesis	13 (8.4%)	56 (36.1%)	<0.001
Del Nido cardioplegia	140 (90.3%)	127 (81.9%)	0.010
Mechanical ventilation, h	8.4 (6.7–12.7)	10.7 (8.0–13.6)	0.002
ICU stay, h	51.9 (46.6–75.6)	54.3 (49.5–76.2)	0.099
Hospital length of stay, days	7 (7–8)	7 (7–9)	0.610

Values are median (interquartile range) or *n* (%) unless otherwise noted. CPB, cardiopulmonary bypass; ICU, intensive care unit.

**Table 3:** Primary and secondary clinical outcomes.

Outcome	miniAVR (*n* = 155)	Conventional (*n* = 155)	*p*-Value
Stroke/TIA (primary)	0 (0.0%)	7 (4.5%)	0.015
30-day mortality	1 (0.6%)	4 (2.6%)	0.371
Overall mortality (full follow-up) ᵈ	7 (4.5%)	17 (11.0%)	0.054
Postoperative atrial fibrillation	21 (13.5%)	16 (10.3%)	0.484
Acute renal failure	2 (1.3%)	2 (1.3%)	1.000
Re-exploration (bleeding/tamponade)	2 (1.3%)	3 (1.9%)	1.000
Permanent pacemaker	1 (0.6%)	4 (2.6%)	0.371
Pneumonia	1 (0.6%)	2 (1.3%)	1.000
Perioperative myocardial infarction	2 (1.3%)	0 (0.0%)	0.498
Pleural drainage ᵉ	5 (3.2%)	3 (1.9%)	0.723

Values are *n* (%). Comparisons by Fisher exact test. ^d^ Median follow-up was 1.9 years (miniAVR) and 4.3 years (conventional); this cumulative count reflects all deaths observed during the entire follow-up and is not directly comparable to the 5-year Kaplan–Meier estimates. ^e^ Referred to as chest-tube drainage in Supplementary Table S3; the two terms denote the same variable. TIA, transient ischemic attack.

**Table 4:** Multivariable Firth penalized logistic regression for stroke/TIA (exploratory).

Covariate	Odds Ratio	95% CI	*p*-Value
miniAVR (vs. conventional)	0.052	0.003–0.832	0.037
CPB time, per minute	1.014	1.001–1.028	0.032
Age, per year	0.975	0.923–1.030	0.369

Firth penalized maximum likelihood logistic regression. Model fit: AIC 61.17; McFadden pseudo-R^2^ 0.205; Hosmer–Lemeshow χ^2^ = 5.16, *p* = 0.523; events-per-variable 2.3 (Supplementary Table S4). CI, confidence interval; CPB, cardiopulmonary bypass.

**Table 5:** Univariable Firth-penalized logistic regression for stroke/TIA.

Covariate	Odds Ratio	95% CI	*p*-Value
miniAVR (vs. conventional)	0.064	0.004–1.135	0.061
CPB time, per minute	1.013	1.002–1.025	0.023
Age, per year	0.958	0.906–1.012	0.123
LV ejection fraction, per %	0.993	0.915–1.079	0.876
GFR, per mL/min/1.73 m^2^	0.998	0.948–1.050	0.930
Atrial fibrillation	0.698	0.037–13.236	0.811
Peripheral vascular disease	1.696	0.081–35.633	0.734

Firth penalized maximum likelihood logistic regression (univariable). CI, confidence interval; CPB, cardiopulmonary bypass; GFR, estimated glomerular filtration rate; LV, left ventricular.

## Data Availability

The data supporting the findings of this study are available from the corresponding author upon reasonable request.

## Supplementary Materials

The following supporting information can be downloaded at: https://www.mdpi.com/article/10.3390/jcm15207803/s1, Table S1: Baseline characteristics before propensity score matching; Table S2: Kaplan–Meier survival estimates and number at risk; Table S3: Continuous secondary outcomes; Table S4: Firth model diagnostics (multivariable); Figure S1: Distribution of propensity scores before and after matching; Figure S2: Forest plot of the primary stroke/TIA endpoint by clinical subgroup.

## Abbreviations

The following abbreviations are used in this manuscript: AF, atrial fibrillation; AIC, Akaike information criterion; AS, aortic stenosis; AVR, aortic valve replacement; BMI, body mass index; CI, confidence interval; CPB, cardiopulmonary bypass; EACTS, European Association for Cardio-Thoracic Surgery; ESC, European Society of Cardiology; EuroSCORE, European System for Cardiac Operative Risk Evaluation; GFR, estimated glomerular filtration rate; ICU, intensive care unit; IQR, interquartile range; KM, Kaplan–Meier; LV, left ventricular; LVEF, left ventricular ejection fraction; miniAVR, minimally invasive aortic valve replacement; OR, odds ratio; PICO(T), Population, Intervention, Comparator, Outcomes, Timing; PSM, propensity score matching; RMST, restricted mean survival time; RR, relative risk; SAVR, surgical aortic valve replacement; SMD, standardized mean difference; STROBE, Strengthening the Reporting of Observational Studies in Epidemiology; TAVI, transcatheter aortic valve implantation; TIA, transient ischemic attack.
