# Supplementary material for: Minimally Invasive Aortic Valve Replacement and Perioperative Stroke Risk: A Propensity-Matched Analysis
Source: J Clin Med. 2026 Oct 10;15(20):7803. doi: 10.3390/jcm15207803 (PMC13705071; doi:10.3390/jcm15207803)
Supplement: Supplementary file 1 [file jcm-15-07803-s001.zip › jcm-4596307-supplementary.pdf]

Supplementary Material

Supplementary Table S1. Baseline characteristics before propensity score matching.

Variable	miniAVR (n=156)	Conventional (n=1,278)	p-value
Age, years	65.9 ± 10.7	71.3 ± 10.9	<0.01
Male sex	90 (57.7%)	761 (59.6%)	0.70
Body mass index, kg/m²	28.0 ± 4.6	28.6 ± 4.7	0.09
LV ejection fraction, %	55.1 ± 8.6	53.2 ± 9.6	<0.01
GFR, mL/min/1.73 m²	79.6 ± 13.7	76.3 ± 15.7	<0.01
EuroSCORE	1.3 ± 0.9	1.7 ± 1.6	<0.01
Hypertension	132 (84.6%)	1,072 (84.0%)	0.94
Diabetes mellitus	31 (19.9%)	352 (27.6%)	0.05
Atrial fibrillation (paroxysmal)	13 (8.3%)	164 (12.9%)	0.14
Cerebrovascular disease	3 (1.9%)	32 (2.5%)	1.00
Bicuspid aortic valve	80 (51.3%)	418 (32.8%)	<0.01
Severe aortic stenosis	134 (85.9%)	1,091 (85.5%)	0.99

Values are mean ± standard deviation or n (%). GFR, estimated glomerular filtration rate; LV, left ventricular.

Supplementary Table S2. Kaplan–Meier survival estimates and number at risk.

Year	miniAVR survival	miniAVR at risk	Conventional survival	Conventional at risk
0	100.0%	155	100.0%	155
1	99.4%	113	96.1%	129
2	—	71	—	113
3	97.8%	32	91.9%	94
4	—	27	—	84
5	94.6%	24	87.2%	64

Log-rank χ^2 = 3.934, p = 0.047. Five-year restricted mean survival time: 4.86 (miniAVR) versus 4.65 (conventional) years. Em dashes (—) indicate years for which a survival percentage was not separately reported; the number-at-risk column is complete for all years shown.

Supplementary Table S3. Continuous secondary outcomes.

Outcome	miniAVR, median (IQR)	Conventional, median (IQR)	p-value
Chest-tube drainage, mL	480 (313–670)	500 (350–735)	0.240
Peak troponin, ng/mL	0.3 (0.2–0.4)	0.2 (0.2–0.4)	0.299
Mechanical ventilation, h	8.4 (6.7–12.7)	10.7 (8.0–13.6)	0.002
ICU stay, h	51.9 (46.6–75.6)	54.3 (49.5–76.2)	0.099

Comparisons by Mann–Whitney U test. ICU, intensive care unit; IQR, interquartile range.

Supplementary Table S4. Firth model diagnostics (multivariable).

Diagnostic	Value
Akaike information criterion (AIC)	61.17
McFadden pseudo-R^2	0.205
Hosmer–Lemeshow χ^2	5.16
Hosmer–Lemeshow p-value	0.523
Events per variable	2.3
Number of events / total	7 / 310

Penalized maximum likelihood estimation (Firth method).

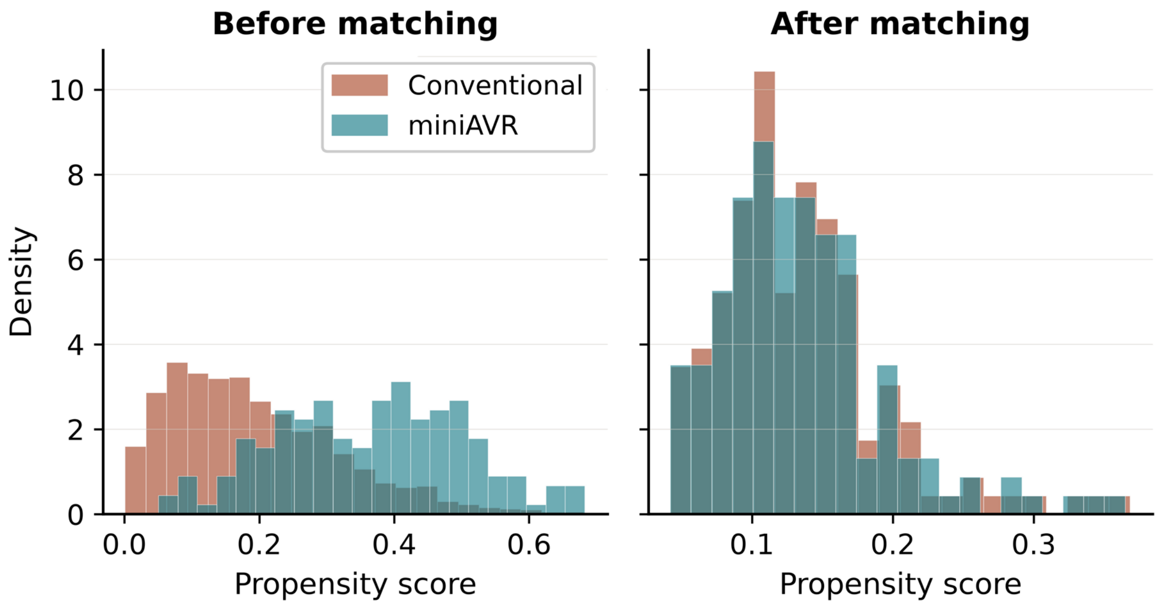

Supplementary Figure S1. Distribution of propensity scores before and after matching, demonstrating substantial overlap in the matched cohort.

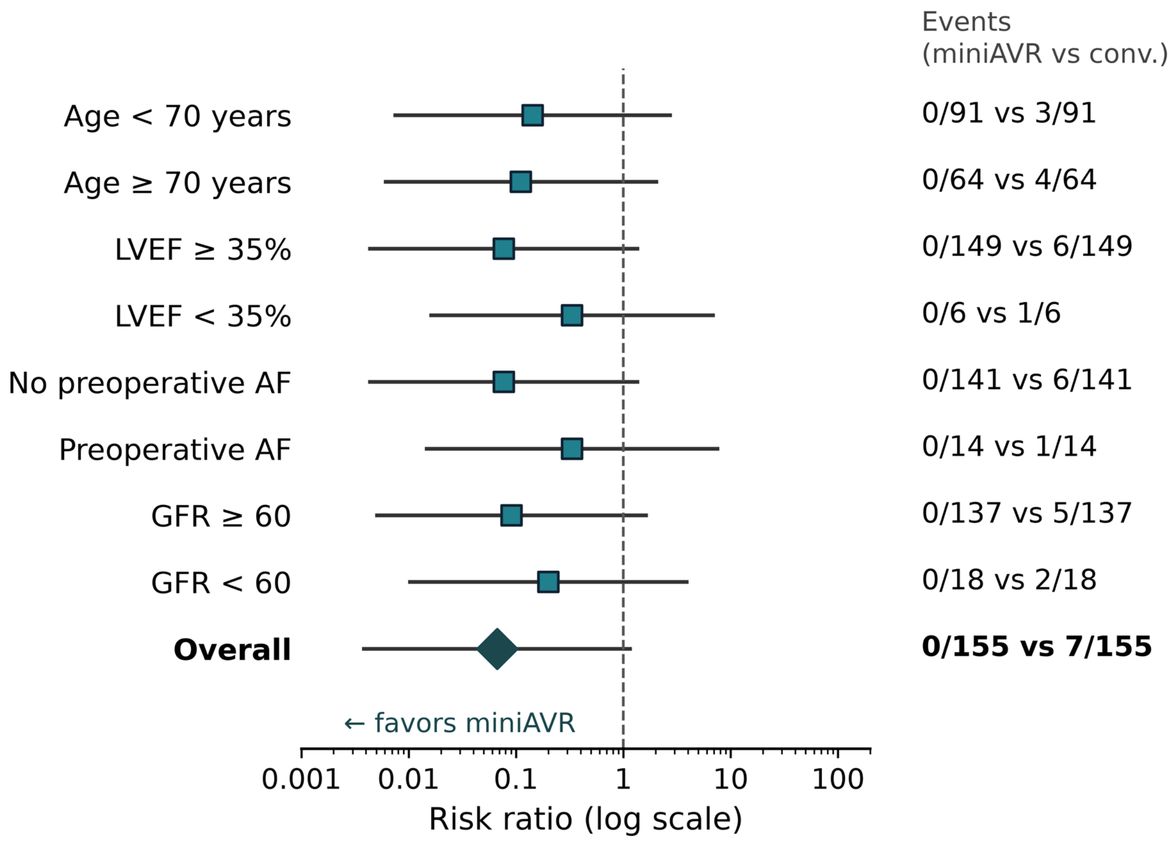

Supplementary Figure S2. Forest plot of the primary stroke/TIA endpoint by clinical subgroup (age, LVEF, preoperative atrial fibrillation, and renal function), expressed as risk ratios with 95% confidence intervals.

Because zero stroke/TIA events occurred in the miniAVR arm in every subgroup, risk ratios and 95% confidence intervals were computed after applying a Haldane–Anscombe continuity correction (0.5 added to each cell) to permit estimation; subgroup point estimates should be interpreted as descriptive given this sparse-data setting.
